# Multi‐Parametric MRI assessment of biomarkers in a non‐human primate model exhibiting sporadic cerebral amyloid angiopathy

**DOI:** 10.1002/alz.092589

**Published:** 2025-01-09

**Authors:** Suleiman Khan, Sean Murray, Jakub Szabo, Muhammad T Soliman, Hannah Goldman, Michael Llanos, Olivia Cho, Emily Schissler, Charles Kingsley, Jody Swain, Stanton B Gray, William D Hopkins, Jelle Veraart, Thomas Wisniewski, Youssef Zaim‐Wadghiri, Henrieta Scholtzova

**Affiliations:** ^1^ NYU Grossman School of Medicine, New York, NY USA; ^2^ Small Animal Imaging Facility, UT MD Anderson Cancer Center, Houston, TX USA; ^3^ UT MD Anderson Cancer Center, Michale E. Keeling Center for Comparative Medicine and Research, Bastrop, TX USA

## Abstract

**Background:**

Cerebral amyloid angiopathy (CAA) has been recognized as one of the morphologic hallmarks of Alzheimer disease (AD). The development of new AD drugs has brought unforeseen challenges that manifest as amyloid‐related imaging abnormalities (ARIA) appearing as vasogenic edema/effusion (ARIA‐E) and cerebral microhemorrhage/hemosiderosis (ARIA‐H). The prominence of CAA pathology in aged squirrel monkeys (SQMs), a New World non‐human primate model, underlines the importance of advancing this unique species for use in AD and dementia research. Previously, we demonstrated the effectiveness of atlas‐based R2* mapping, a measure of transverse tissue relaxation rate sensitive to the presence of iron, to discern age‐associated changes in SQM neuropathology along with validation of multiparametric in‐vivo MRIs to identify spontaneous ARIA in geriatric SQMs. The present study examined SQMs at distinct stages of aging by combining quantitative R2* mapping, T_2_‐weighted (T_2_‐w) sequences, and multi‐shell diffusion weighted imaging (DWI) to evaluate microstructural tissue integrity, in addition to histological assessment of the brain pathology correlates.

**Method:**

SQMs underwent 2D T_2_‐w Turbo Spin Echo (TSE) RARE/FLAIR MRI scans, in addition to multi‐shell DWI acquisitions. In vivo multi‐gradient echo (MGE) scans across three age cohorts were utilized to generate R2* maps for atlas‐based quantitative assessments of neuropathology. Coronal brain sections were examined to characterize histopathological features of MRI lesions.

**Result:**

Multiple instances of ARIA‐E‐like signal hyperintensities have been identified in brains of geriatric SQMs using T_2_‐w TSE RARE/FLAIR and DWI MRI. Furthermore, DWI revealed white matter (WM) abnormalities undetectable by conventional MRI. Absolute R2* values significantly increased with age in several brain regions, both in GM and WM. However, some regions experienced a reversal of this trend, suggesting a multi‐factorial model. Histological assessment of brain tissue corresponding to MRI abnormalities/hyperintensities revealed extravascular fibrinogen, diffuse IgG deposits, reactive gliosis, and disruptions of axons and myelin. In ARIA‐H regions, Perls’ stain confirmed hemosiderin depositions surrounding CAA‐positive blood vessels. Ferritin staining revealed microglial cells with characteristic morphology alongside colocalization of iron within microglia cell bodies.

**Conclusion:**

Overall, this study demonstrates the complex interplay between ARIA, CAA, and age‐related brain changes in SQMs, highlighting the utility of this model for translational research on CAA/AD and innovative therapeutic interventions.

